# Osmotic stress responses and the biology of the second messenger c-di-AMP in *Streptomyces*

**DOI:** 10.1093/femsml/uqad020

**Published:** 2023-04-11

**Authors:** Sukanya Bhowmick, Mary L Shenouda, Natalia Tschowri

**Affiliations:** Institute of Microbiology, Leibniz Universität Hannover, 30419 Hannover, Germany; Institute of Microbiology, Leibniz Universität Hannover, 30419 Hannover, Germany; Institute of Microbiology, Leibniz Universität Hannover, 30419 Hannover, Germany

**Keywords:** *Streptomyces*, osmotic stress, DisA, AtaC, c-di-AMP

## Abstract

*Streptomyces* are prolific antibiotic producers that thrive in soil, where they encounter diverse environmental cues, including osmotic challenges caused by rainfall and drought. Despite their enormous value in the biotechnology sector, which often relies on ideal growth conditions, how *Streptomyces* react and adapt to osmotic stress is heavily understudied. This is likely due to their complex developmental biology and an exceptionally broad number of signal transduction systems. With this review, we provide an overview of *Streptomyces*' responses to osmotic stress signals and draw attention to open questions in this research area. We discuss putative osmolyte transport systems that are likely involved in ion balance control and osmoadaptation and the role of alternative sigma factors and two-component systems (TCS) in osmoregulation. Finally, we highlight the current view on the role of the second messenger c-di-AMP in cell differentiation and the osmotic stress responses with specific emphasis on the two models, *S. coelicolor* and *S. venezuelae*.

## Teaser

In this review, we summarize how antibiotic producing Streptomyces respond to osmotic challenges with a focus on c-di-AMP signalling pathways that contribute to stress adaptation of these bacteria.

## Introduction

One of the most ubiquitous environmental stresses encountered by microorganisms in their natural habitats are changes in the external osmotic conditions. In particular, soil bacteria are often exposed to drastic changes caused by rainfall or drought and have evolved clever strategies to adapt to variations in osmolality. Cellular cytoplasm contains high concentration of various solutes and constituents causing an osmotic pressure, the turgor, which is essential for all cells encased in a cell wall (Wood 2011). Because of the semipermeable nature of the cytoplasmic membrane, water influx or efflux is triggered by changes in the osmolality of the environment, affecting the osmotic potential of the cytoplasm and the vitality of the cell (Wood 1999). To control transmembrane water fluxes, bacteria modulate their cytoplasmic composition through the accumulation or expulsion of osmolytes. They respond to external osmotic upshifts by increasing the cellular concentration of cations (e.g. K^+^) and compatible solutes such as amino acids (e.g. proline), amino acid derivatives (e.g. ectoine), and oligosaccharides (e.g. trehalose), which consequently increases the osmotic potential of the cytoplasm, restricts water efflux, promotes water influx, and hinders cell shrinkage and collapse. On the contrary, under a sudden drop in the environmental osmolality, bacteria release ions and compatible solutes from the cell to limit water influx, which may cause cell swelling and, in the worst cases, cell burst (Bremer and Krämer 2019).

Osmolyte transport systems and compatible solutes biosynthesis pathways play a central role in the cellular adaptation to osmotic stress and are well studied in the rod-shaped model species *Escherichia coli, Bacillus subtilis*, and *Corynebacterium glutamicum* (Krämer 2009, Wood 2011, Hoffmann and Bremer 2017). However, our knowledge about mechanisms involved in osmo-sensing and -responding in bacteria of the genus *Streptomyces* is very fragmented, despite their enormous value for the medical and biotechnological sectors and their important contribution to healthy soil ecology. Streptomycetes are members of Gram-positive Actinobacteria that produce a broad range of bioactive compounds such as antibiotics, anti-cancer compounds, immunosuppressants, and anti-parasitic substances, and hence represent a key source for drugs to combat diseases (Hopwood 2007, van Bergeijk et al. 2020). They are highly abundant in the soil habitat, where they secrete a remarkable repertoire of hydrolytic enzymes that are essential for recycling of organic matter in nature and, in addition, are extensively used in biotechnology (Chater et al. 2010, Spasic et al. 2018).

In this review, we summarize current knowledge about how streptomycetes react to osmotic stress conditions, focussing on responses specific for a filamentous model and elucidating the molecular links between osmostress signalling and cell differentiation with particular emphasis on the roles of c-di-AMP signalling cascades.

### Osmostress signals trigger morphological changes in *Streptomyces*

In streptomycetes, the pattern of their filamentous growth and cell differentiation are affected by osmotic challenges. These bacteria are characterized by astonishing morphological plasticity and undergo a complex transition from filamentous vegetative hyphae to spores during their developmental life cycle. The vegetative mycelium consists of long, multicellular filaments that scavenge for nutrients and grow by tip extension and through initiation of new branches behind the tip (Flärdh and Buttner 2009, Flärdh et al. 2012). Apical growth requires the localization of peptidoglycan synthases, hydrolases, and other proteins involved in assembly of the cell wall to one cell pole and is directed by the cytoskeletal-like coiled-coil protein DivIVA (Flärdh 2003). In *Streptomyces*, DivIVA forms discrete foci at growing tips and together with other proteins, such as Scy and FilP, constitutes the polarisome that guides cell polarity (Holmes et al. 2013, Frojd and Flärdh 2019a). Splitting of the polarisomes at growing tips gives rise to daughter polarisomes that coordinate the emergence of new branches upon reaching a critical size (Hempel et al. 2012, Richards et al. 2012). The Ser/Thr protein kinase AfsK co-localizes with DivIVA and phosphorylates several serine and threonine residues in DivIVA in response to stress signals that compromise the cell wall biosynthetic machinery, such as bacitracin or vancomycin. On the other hand, the phosphatase SppA dephosphorylates DivIVA *in vivo* and *in vitro* (Passot et al. 2022). High level of DivIVA phosphorylation due to constitutive AfsK kinase activity induces disassembly of the apical polarisome and stimulates the formation of multiple new polarisomes, causing a hyperbranching phenotype (Hempel et al. 2012).

**Figure 1:**
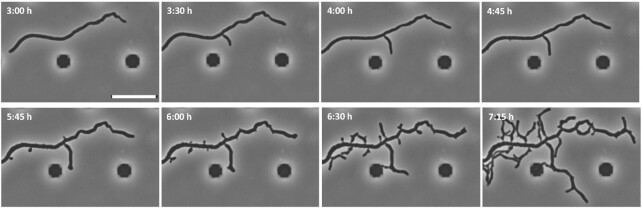
Osmotic upshift causes growth arrest and reprogramming of cell polarity in *S. venezuelae*. Cells were grown for 4 hours in maltose-yeast extract-malt extract medium (MYM) in a microfluidic device and were then subjected to MYM + 0.5 M NaCl. After a growth arrest for about 2 hours, growth recommences from new lateral branching nodes, leading to hyperbranching. Images were taken at the indicated time points using the Thunder Imager Live Cell from Leica. Scale bar: 5 μm.

Exposure of *S. coelicolor* to hyperosmotic stress by addition of either non-ionic osmolyte sucrose or ionic solute NaCl in a microscope growth chamber revealed that osmotic upshift leads to a growth arrest for 2–3 hours. After this adaptive lag phase, the bacteria resume growth but completely restructure their cell polarity and the growth pattern of the mycelium. They form multiple new branches at the lateral sites, leading to hyperbranching mycelium reminiscent of a mutant expressing constitutively active AfsK kinase (Fig. 1) (Hempel et al. 2012, Fuchino et al. 2017). Neither Scy, FilP, nor AfsK seem to be needed for reprogramming of cell polarity after osmotic upshift since the corresponding mutants show a similar response as wildtype *S. coelicolor*. As shown by localization analysis, DivIVA appears to persist at the main hyphal tips after the arrest of tip extension, so that the absence of DivIVA does not account for growth attenuation at the central tip. A similar experimental setup was used to study osmotic downshift, showing that hyphal growth was ceased for 30 min. In contrast to high-osmolality conditions, *S. coelicolor* resumed growth at the existing tips, and no hyperbranching was observed. In *S. venezuelae*, osmotic upshift elicited similar morphological changes, suggesting that dramatic reprogramming of cell polarity could be a conserved response mechanism in different *Streptomyces* species; however, the underlying mechanism remains unknown (Fuchino et al. 2017).

Yet another response of filamentous Actinobacteria to high osmolality is the formation of cell wall-deficient cells. *Kitasatospora viridifaciens* produces viable DNA-containing vesicles at hyphal tips, the so-called S-cells (stress-induced cells), in the presence of both, ionic (NaCl) and non-ionic osmolytes (sorbitol) after apical growth arrest (Ramijan et al. 2018). Vesicle release from hyphal tips upon growth arrest has also been reported for *S. venezuelae*, both under hyperosmotic conditions (Ramijan et al. 2018) and occasionally under non-stress conditions after spontaneous cessation of growth (Frojd and Flärdh 2019b). No such cell wall-deficient cells were found when *S. coelicolor, S. griseus*, and *S. lividans* were used as models. S-cells exposed to hyperosmotic stress for a prolonged period of time accumulate mutations that enable them to grow as wall-deficient cells, the so-called L-forms (Leaver et al. 2009, Ramijan et al. 2018). It is hypothesized that the ability to form S-cells improves fitness in filamentous actinomycetes under hyperosmotic stress; however, it remains to be elucidated how this morphological switch is regulated and how it affects bacterial fitness (Ramijan et al. 2018).

For reproduction, *Streptomyces* raise long, aerial hyphae into the air that give a colony grown on agar plates a white and hairy appearance (Flärdh and Buttner 2009). To escape the surface tension, aerial hyphae and spores are encased in a hydrophobic sheath mainly consisting of chaplin and rodlin proteins (Claessen et al. 2003, Elliot et al. 2003). *S. venezuelae* secretes two long (ChpB and ChpC) and five short (ChpD-H) chaplins, and these proteins are expected to self-assemble into amyloid-like filaments on the cell surface (Bibb et al. 2012). Rodlin proteins (RdlA-C) are proposed to organize the chaplin filaments into so-called rodlets, but seem to be dispensable for aerial development and surface hydrophobicity under normal growth conditions (Claessen et al. 2003). Moreover, *Streptomyces* secrete an additional surfactant, the lantibiotic-like peptide SapB, the product of *ramS* encoded in the *ramCSAB* operon (Willey et al. 1991, Kodani et al. 2004).

**Figure 2:**
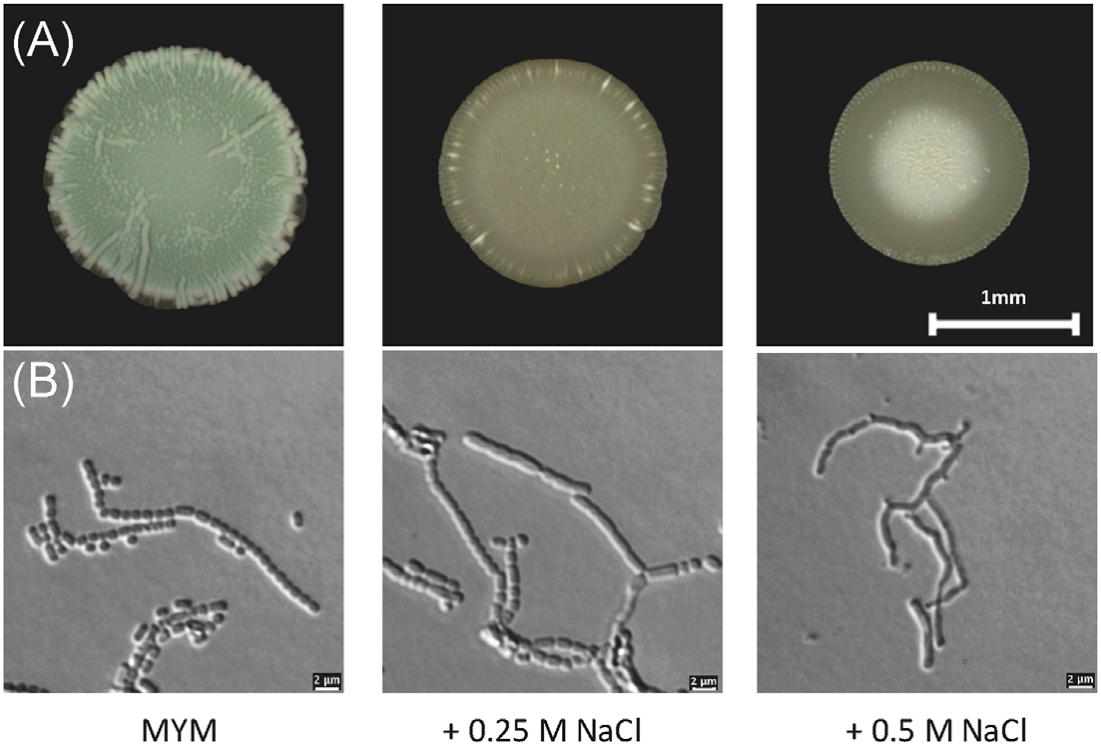
Effect of osmotic stress on the developmental progression of *S. venezuelae*. (A) Macrocolonies of wildtype *S. venezuelae* were grown on solid sporulation media (MYM agar) without added salt and either supplemented with 0.25 or 0.5 M NaCl and incubated at 30°C for 2 days. Images were taken using S9 i stereomicroscope from Leica. (B) Differential contrast interference (DIC) microscopy images from the cover slip imprints of the macrocolonies showing that the wildtype *S. venezuelae* forms spores of regular size and shape that are organized in chains. Addition of 0.25 M NaCl causes defects in spore formation. Further increase of added NaCl to 0.5 M completely blocks sporulation. Images were taken using the Thunder Imager Live Cell from Leica.

The final morphological transition involves the synchronized division of the aerial hyphae into chains of spores, which is mainly driven by the GTPase FtsZ that polymerizes into filaments, the Z-rings, and recruits additional cell division proteins (Jakimowicz and van Wezel 2012, Bush et al. 2015). Mature spores accumulate pigments that are frequently aromatic polyketides produced by enzymes encoded in the highly conserved *whiE* cluster (Kelemen et al. 1998). Pigmentation gives the *Streptomyces* colony a characteristic colour, e.g. mature *S. venezuelae* colonies appear green to the eye (Fig. 2A).

Addition of salt to the growth medium strongly inhibits morphological differentiation in both, *S. coelicolor* (Sevcikova and Kormanec 2004) and *S. venezuelae* (Fig. 2). In the presence of high NaCl, *S. venezuelae* fails to raise aerial hyphae, which is why the colony remains bald and shiny. In comparison, unstressed cells become green after the same incubation time, indicating that mature spores have been produced out of aerial hyphae (Fig. 2A). Despite failures in the formation of aerial filaments, *S. venezuelae* produces spores at 0.25 M NaCl; however, further increase of NaCl concentration to 0.5 M seems to completely block spore formation (Fig.2B). Salt stress-induced inhibition of sporulation seems to be counterintuitive considering that the formation of resistant spores might be an escape route when vegetative growth becomes challenging. However, *B. subtilis* seems to apply the same strategy and also blocks sporulation under salt stress. The authors propose that at high ionic osmolality conditions, *B. subtilis* would not be able to complete the time- and energy-consuming sporulation programme and therefore blocks entry into sporulation in high-salinity environments (Widderich et al. 2016). A similar but yet unproven logic may apply for *Streptomyces*.

The hydrophobic coat components chaplins, rodlins, and SapB are important for cell differentiation under osmotic upshift conditions. *S. coelicolor* strains defective in SapB biosynthesis due to *ramS* or *ramR* deletion, show a delay in development in the presence of 10.3% sucrose or 500 mM KCl. Similarly, development of strains lacking the rodlins RdlA and RdlB or of the *chpABCDH* mutant, missing five of the eight *S. coelicolor* chaplin genes, is delayed (de Jong et al. 2012). In *S. venezuelae*, expression of *ramS* (*vnz_31970*) is ∼6-fold downregulated on nutrient agar containing 0.5 M NaCl when compared to medium without extra added salt (Sukanya Bhowmick and Natalia Tschowri, unpublished data). Reduced production of SapB under such conditions likely compromises aerial mycelium formation, as shown in Fig. 2A.

Biosynthesis of natural products is triggered by a wide variety of environmental and physiological signals and is genetically and temporally connected to the developmental biology of streptomycetes (Bibb 2005). Genes that are responsible for the biosynthesis of an individual secondary metabolite are usually clustered together to form a biosynthetic gene cluster (BGC). Most of these BGCs are regulated by pathway-specific regulatory genes that are located within the cluster. These pathway-specific regulators are usually controlled by other pleiotropic regulators, and therefore, different environmental and physiological stresses can lead to changes in the levels of production of different secondary metabolites (Bibb 2005, Liu et al. 2013). *S. coelicolor* produces two pigmented antibiotics: the blue actinorhodin (ACT) and the red undecylprodigiosin (RED). The synthesis of ACT is dependent on the pathway-specific regulator ActII-ORF4, which activates the expression of genes that encode biosynthetic enzymes within the *act* gene cluster (Fernandez-Moreno et al. 1991). On the other hand, RedD is required for activation of the RED biosynthetic genes (Rudd and Hopwood 1980). High salt conditions inhibit ACT production but have a stimulatory effect on RED biosynthesis. The effects on antibiotic biosynthesis are mediated at the transcriptional level since salt has been shown to inhibit *actII-ORF4* and to stimulate *redD* expression, respectively (Sevcikova and Kormanec 2004). In the following sections, we elaborate on the molecular components and mechanisms that affect the phenotypes associated with adaptation to osmotic stress and discuss potentially involved potassium transport systems, compatible solutes, the role of two-component systems (TCS), alternative sigma factors, and c-di-AMP.

### Potassium transport systems and their role in osmoadapation

A key aspect of microbial adaptation to changes in environmental osmolality is to adjust and direct water fluxes across their membrane to prevent cell dehydration at high external osmolality and cell burst at low external osmolality. Biosynthesis, uptake, and export of compatible solutes play a central role in these adjustment processes but are, with few exceptions, not well studied in *Streptomyces*.

Bacteria react to a sudden drop in the environmental osmolality by transient opening of mechanosensitive channels of the MscL (L for large) or MscS (S for small) type, which generally are non-selective in terms of the ions and molecules that pass through the pore (Booth 2014). This is an emergency reaction essential for preventing turgor increase to a degree that may cause cell rupture, as sudden osmotic downshift causes immediate water influx (Buda et al. 2016). Little is known about mechanosensitive channels in *Streptomyces*; however, our *in silico* analysis revealed that *S. venezuelae* contains two large (Vnz_14925 and Vnz_21315) and two small (Vnz_00645 and Vnz_11950) conductance mechanosensitive channels, while *E. coli*, e.g. possesses one MscL and six MscS-like channels (Edwards et al. 2012). A study addressing the physiological function of SC-MscL (SCO3190) in *S. coelicolor*, suggests that SC-MscL can contribute to the secretion of antibiotics since overexpression of the corresponding gene was shown to result in increased secretion of the blue antibiotic ACT and to affect colony size (Wang et al. 2007). In addition to mechanosensitive channels, many bacteria also possess water-specific channels, the aquaporins, which accelerate water fluxes across the membrane (Delamarche et al. 1999). *S. venezuelae* possesses two aquaporins (Vnz_11070 and Vnz_17040), characterized by the aquaporin transporter signature IPR034294, and one aquaporin-like protein, Vnz_06120, having the IPR023271 domain. However, so far, the precise role of aquaporins in microbial osmotic stress response is not well understood (Tanghe et al. 2006, Akai et al. 2012).

Temporal import of ions, in particular that of K^+^, is an emergency reaction of many bacteria upon a sudden osmotic upshift to limit water efflux (Wood 2011). For example, *B. subtilis* increases the K^+^ pool from about 350–700 mM within 1 hour (minimal medium, 25°C), when exposed to 400 mM NaCl (Whatmore et al. 1990, Hoffmann and Bremer 2017). For *S. griseus*, a sharp increase of intracellular potassium was reported when exposed to NaCl concentrations >0.75 M, whereas internal sodium concentrations were maintained at very low levels (Killham and Firestone 1984). Every bacterium typically possesses several potassium uptake and release systems that differ in their molecular architecture, activation mechanisms, affinity for potassium and transport rates, and type of ion translocation system. Passive channels allow K^+^ flow down the electrochemical gradient without any energy input, while active transporters use ATP or the proton motive force for potassium accumulation (Stautz et al. 2021).

The only studied K^+^ transport system in the genus *Streptomyces* is the potassium channel KcsA, which was discovered in *S. lividans* in 1995 and since then has served as an extensively studied model for pro- and eukaryotic potassium channels (Schrempf et al. 1995). Structural analysis of KcsA revealed that the channel is tetrameric, each monomer containing two transmembrane helices and a pore domain between them (Doyle et al. 1998). KcsA is voltage-dependent, highly selective for potassium, and activated by a downshift of intracellular pH (Hirano et al. 2011). Despite detailed biochemical and structural analysis of KcsA, its physiological function in the biology of streptomycetes is unknown.

Aiming to identify the full set of potassium transporters in the model organism *S. venezuelae*, we searched for putative potassium transporters using the TransportDB database (Elbourne et al. 2017) and by using the BLAST function in StrepDB (https://strepdb.streptomyces.org.uk/) and known K^+^-translocating systems described in Stautz *et al*. (2021) as a reference. This is not an easy task considering that *S. venezuelae* contains an impressive number of 756 transport proteins, as predicted by TransportDB (Elbourne et al. 2017). However, as summarized in Table 1, we found nine putative potassium transporters, of which none have been experimentally studied yet. This set includes the active, high-affinity K^+^-dependent P-Type ATPase KdpFABC, encoded by the *vnz_12290–12275* genes. It is a heterotetrameric K^+^ pump that has been best characterized in *E. coli* (Bramkamp et al. 2007). It consists of the P-type ATPase KdpB, KdpA belonging to the superfamily of K^+^ transporters, and the supplementary proteins KdpF and KdpC (Huang et al. 2017). We also found two KimA-like K^+^/H^+^ symporters of the KUP family that share 39% (Vnz_27465), and 42% (Vnz_15480), respectively, identical residues with KimA from *B. subtilis* (Gundlach et al. 2019, Tascon et al. 2020). KimA, containing 12 transmembrane helices and a cytosolic domain, forms a homodimer in the membrane. In *B. subtilis*, it has a medium affinity for K^+^ and is inactivated by the second messenger cyclic di-3′,5′-adenosine monophosphate (c-di-AMP) (Stülke and Krüger 2020, Tascon et al. 2020). Two proteins (Vnz_28525 and Vnz_28050/CpeB) show 34% and 32% identity, respectively, to the K^+^/H^+^ antiporter KhtU. The transport system consists of the membrane protein KhtU and the cytosolic subunit KhtT and is activated by the binding of c-di-AMP to the C-terminal RCK_C (regulator of conductance of K^+^, C-terminal) domain in KhtT (Schrecker et al. 2019, Cereija et al. 2021). The potential role of c-di-AMP in the regulation of CpeB function in *S. venezuelae* will be discussed below. Finally, we found four putative potassium channels carrying the ‘potassium channel domain IPR013099’, namely Vnz_17705, Vnz_00420, Vnz_05495, and Vnz_10685 (Table 1). Of these, Vnz_17705 shares 24% identical residues with the Ca^2+^-gated potassium channel MthK from *Methanothermobacter thermautotrophicus* (Jiang et al. 2002). Of note, we could not find KcsA or any other known potassium transporters described in Stautz et al. (2021) such as TrkH, KtrB, KtrD, YugO, or Kch in *S. venezuelae*.

**Table 1.** Putative potassium transporters in *S. venezuelae*. Candidates for potassium uptake and/or export were identified using TransportDB database (Elbourne et al. 2017) and the BLAST function in StrepDB (https://strepdb.streptomyces.org.uk/). None of the listed proteins has been characterized experimentally prior publication of this review. Relevant domains were identified using InterPro (https://www.ebi.ac.uk/interpro/). Number of transmembrane domains (TM) was predicted using uniprot (https://www.uniprot.org/). For details see text.

vnz number (StrepDB)	sven number (StrepDB)	Name	Predicted domain architecture	Family
Vnz_12290	Sven2511	KdpF	1 TM helix	K^+^-transporting ATPase, subunit F
Vnz_12285	Sven2510	KdpA	10 TM helices	K^+^-transporting ATPase, subunit A
Vnz_12280	Sven2509	KdpB	7 TM helices P-type ATPase, cytoplasmic domain N (IPR023299); P-type ATPase, phosphorylation site (IPR018303); P-type ATPase, A domain superfamily (IPR008250)	K^+^-transporting ATPase, subunit B
Vnz_12275	Sven2508	KdpC	1 TM helix	K^+^-transporting ATPase, subunit C
Vnz_27465	Sven5551		12 TM helices Amino acid/polyamine transporter I (IPR002293)	Conserved membrane protein (39% identity to KimA from *B. subtilis*)
Vnz_15480	Sven3148		12 TM helices Amino acid/polyamine transporter I (IPR002293)	Conserved membrane protein (42% identity to KimA from *B. subtilis*)
Vnz_28525	Sven5767		13 TM helices Cation/H+ exchanger (IPR006153)	Putative transmembrane transport protein (34% identity to KhtU from *B. subtilis)*
Vnz_28050	Sven5672	CpeB	13 TM helices Cation/H+ exchanger (IPR006153)	Putative transmembrane transport protein (32% identity to KhtU from *B. subtilis)*
Vnz_17705	Sven3600		2 TM helices Potassium channel domain (IPR013099) NAD(P)-binding domain superfamily (IPR036291) Regulator of K+ conductance, N-terminal (IPR003148)	Potassium channel protein (24% identity to the Ca^2+^ gated potassium channel MthK from *M. thermautotrophicus*)
Vnz_00420	Sven0095		3 TM helices Potassium channel domain (IPR013099)	Potassium channel protein
Vnz_05495	Sven1132		4 TM helices Potassium channel domain (IPR013099) Voltage-gated potassium channel (IPR028325)	Potassium voltage-gated channel subfamily KQT
Vnz_10685	Sven2186		2 TM helices Potassium channel domain (IPR013099) Voltage-gated potassium channel (IPR028325)	Potassium voltage-gated channel subfamily KQT

### Compatible solutes in *Streptomyces*’ osmoadaptation

Shortly after bacteria respond to external osmotic upshifts through the rapid import of potassium ions, they begin to synthesize or import compatible solutes and reduce the ionic strength of the cytoplasm through the export of K^+^ (Empadinhas and da Costa 2008). Compatible solutes are water-soluble, osmotically active organic compounds, which are mostly amino acids and carbohydrates or their derivatives that provide osmotic balance without interfering with cellular physiology and biochemistry. Bacteria use different combinations of compatible solutes, depending on their physiology and the level of salinity of their growth habitat, e.g. *B. subtilis* uses 15 different osmostress protectants (Sleator and Hill 2002, Hoffmann and Bremer 2017). An early study analysing the physiological response of *S. griseus* and *S. californicus* to challenges with NaCl or KCl (0.25–1 M) reported that the concentration of proline, glutamine, and alanine dramatically increased in cells. The highest degree of accumulation was detected for proline, which increased from <6% of the free amino acid pool in cells grown in basal medium to about 50% of the pool in cells stressed with 1 M salt (Killham and Firestone 1984). Global metabolomic characterization of the salt response in *S. coelicolor* revealed that this model species also strongly accumulates proline; however, an increase of arginine, phenylalanine, methionine, tryptophan, and (iso)leucine has also been reported (Kol et al. 2010). Thus, free amino acids, especially proline, play an important role in osmoprotection in streptomycetes.

In addition to free amino acids, the capacity to synthesize the tetrahydropyrimidine derivative ectoine as a compatible solute is widespread in streptomycetes (Bursy et al. 2008, Pastor et al. 2010). Ectoine was discovered in the extremely halophilic bacterium *Halorhodospira halochloris* (formerly *Ectothiorhodospira halochloris*) (Galinski et al. 1985), followed by the discovery of its hydroxylated derivative 5-hydroxyectoine in *S. parvulus* (Inbar and Lapidot 1988). The ectoine BGC comprises the *ectABCD* genes, which encode for the L-2,4-diaminobutyrate transaminase EctB, the L-2,4-diaminobutyrate acetyltransferase EctA, the ectoine synthase EctC, and the ectonie hydroxylase EctD and was studied in *S. coelicolor* (Bursy et al. 2008, Kol et al. 2010, Czech et al. 2018). 5-hydroxyectoine was found to accumulate strongly in *S. coelicolor* in response to salt exposure, indicating that it acts as a key osmoprotectant (Bursy et al. 2008, Kol et al. 2010). Growth analysis of single *S. coelicolor ectA, ectC*, or *ectD* mutants in the presence of 1 M NaCl showed that disruption of either *ectA* or *ectC* leads to a salt-sensitive phenotype, which can be complemented by addition of ectoine/5-hydroxyectoine to the medium (Bursy et al. 2008, Kol et al. 2010). Thus, *S. coelicolor* possesses an uptake system for ectoine/5-hydroxyectoine, but the identity of this transporter has not yet been identified. In addition to their osmoprotective function, ectoine and hydroxyectoine were also reported to act as chemical chaperones (Czech et al. 2018). They are capable of protecting macromolecules such as enzymes and nucleic acids against different stress conditions such as heat, cold, and UV stress. Therefore, these compounds have found different applications in industry as stabilizers of proteins and cells in life sciences and in cosmetics (Pastor et al. 2010, Hermann et al. 2020).

Finally, various strains of *Streptomyces* have been reported to contain high levels of the disaccharide trehalose in their spores (Hey-Ferguson et al. 1973, Braña et al. 1986, Rueda et al. 2001). In *S. antibioticus*, trehalose content was reported to increase during development. Vegetative hyphae contained 2%, aerial hyphae 5%, and spores 12% of total dry cell weight (Braña et al. 1986). *De novo* trehalose biosynthesis in *Streptomyces* depends on the trehalose-6-phosphate synthase OtsA, which uses GDP-glucose as the donor substrate, and the trehalose-6-phosphate phosphohydrolase OtsB (Asencion Diez et al. 2017). In addition, the glucose storage compound glycogen, which accumulates in sporulating hyphae, can be converted by TreY/TreZ to trehalose during spore maturation (Schneider et al. 2000, Rueda et al. 2001, Schumacher et al. 2022). Trehalose is present in both prokaryotic and eukaryotic organisms, including bacteria, yeast, and fungi, where it fulfils protective roles for proteins and cellular membranes against desiccation, dehydration, heat, cold, and oxidation (Elbein et al. 2003, Iturriaga et al. 2009). In *E. coli*, it serves as a compatible solute that provides osmoprotection to withstand osmotic stress generated by the addition of 0.5 M NaCl to minimal medium (Strom and Kaasen 1993). In streptomycetes, trehalose is degraded during germination and provides protection against dehydration stress (Hey-Ferguson et al. 1973, Braña et al. 1986, McBride and Ensign 1990); however, whether it contributes to adaptation under changes in external osmolality has not been fully elucidated yet.

### Regulation of cellular responses to osmotic stress

Cellular adjustments to sustained changes in osmolality involve alterations in the gene transcription profile. The best-studied mechanisms for osmostress sensing and regulation are TCS, which typically consist of a membrane-bound sensor histidine kinase and a response regulator (Stock et al. 2000). In *E. coli*, osmoresponsive TCSs include KdpDE, consisting of the membrane-bound histidine kinase KdpD and the response regulator KdpE, which controls the expression of the *kdpFABCDE* operon, encoding the active K^+^ uptake system (see above) and the TCS (Laermann et al. 2013). Another prominent osmosensitive TCS in *E. coli* is EnvZ/OmpR, which regulates the expression of *ompC* and *ompF* genes, encoding two outer membrane porin proteins, in response to external osmolality (Leonardo and Forst 1996). Dissecting specific TCS contributing to osmostress resistance in *Streptomyces* is challenging considering that, e.g. *S. venezuelae* contains 58 TCS operons and additional 27 orphan sensor kinases and 18 orphan response regulator genes (McLean et al. 2019). Of these, only the *osaA/B/C* genes, which are encoded next to each other but are not transcriptionally coupled (Bishop et al. 2004), have been associated with regulation of the osmotic stress response. OsaA (Vnz_26710) and OsaC (Vnz_26705) are hybrid proteins containing both, a histidine kinase and a response regulator receiver domain, while OsaB (Vnz_26715) represents a response regulator of the CheY-type (McLean et al. 2019). Deletion of *osaB* either in *S. lividans* or *S. coelicolor* was reported to compromise aerial mycelium formation when the bacteria were grown on R2YE medium with 10.3% sucrose or on MS agar containing 250 mM KCl. Deletion of *osaA* in *S. coelicolor* had less pronounced consequences and led to only a minor delay in aerial mycelium formation under high osmolality conditions (Bishop et al. 2004). *S. coelicolor* ∆*osaB* has also been shown to have a severe growth defect on SMMS (supplemented minimal medium) containing 1 M NaCl, which could not be rescued by the external addition of ectoine/5-hydroxyectoine (Kol et al. 2010). Similarly, deletion of *osaC* in both, *S. coelicolor* and *S. avermitilis* led to a conditionally bald phenotype since the corresponding mutants failed to raise aerial hyphae under hyperosmotic stress conditions (Fernandez Martinez et al. 2009, Godinez et al. 2015). The study by Fernández Martinez *et al*. (2009) provides few mechanistic insights into the function of the Osa system in *S. coelicolor*. The authors showed that *osaB* expression is strongly upregulated 60 min after osmotic upshift in a σ^B^-dependent manner, but returns to normal levels after about 12 hours of growth. However, in the *osaC* mutant, expression of σ^B^-dependent genes, including *osaB*, remained at relatively high levels. Subsequent interaction analysis revealed that OsaC interacts with σ^B^ via its N-terminal kinase domain. Based on these data, the authors propose that OsaC modulates σ^B^-activity to return expression of the σ^B^ regulon to pre-osmotic stress levels (Fernandez Martinez et al. 2009). Notably, the osmotic stress response in *S. coelicolor* is mediated by multiple sigma factors and involves induction of the stress sigma factor σ^B^ as well as of at least eight other sigma factor genes (*sigL, sigM*, s*igH, sigI, sigJ, sigX, hrdD*, and *hrdB*) (Viollier et al. 2003, Lee et al. 2005). Altogether, these data indicate that OsaA/B/C and a complex sigma factors network likely fulfil a conserved function in adaptation to hyperosmotic stress in the genus *Streptomyces*, but mechanistic details remain poorly defined.

In *C. glutamicum*, the MtrAB TCS responds to hyperosmotic stress and regulates expression of both, genes involved in cell wall biosynthesis and genes encoding compatible solutes carriers, *betP, proP*, and *lcoP*, as well as the *mscL* gene, encoding a mechanosensitive channel (Krämer 2009, Moker et al. 2004). MtrAB is highly conserved throughout the phylum Actinobacteria (McLean et al. 2019). The system has been studied in *S. coelicolor* and *S. venezuelae* and reported to regulate expression of antibiotic genes as well as developmental processes, but has not been linked to the osmotic stress response in streptomycetes yet (Som et al. 2017a, 2017b, Zhang et al. 2017). Similarly, the KdpDE TCS is widely conserved in the genus *Streptomyces* (McLean et al. 2019), but has not been analysed in any model of the genus yet. Our unpublished data indicate that the KdpDE TCS is neither required for *S. venezuelae* development on standard sporulation medium nor for growth on nutrient agar containing 0.5 M NaCl or KCl (Timo Holdgrewe and Natalia Tschowri, unpublished data).

### C-di-AMP signalling network in *Streptomyces*

The nucleotide-based second messenger c-di-AMP is recognized as an important regulator of ion and osmolyte transport to maintain osmotic homeostasis in bacteria and is predominantly found in Gram-positive bacteria and archaea (Corrigan and Grundling 2013, Stülke and Krüger 2020). Diadenylate cyclases (DACs) carrying the enzymatically active DAC domain synthesize c-di-AMP from two molecules of ATP. On the other hand, phosphodiesterases (PDEs) degrade the cyclic molecule to the linear 5´-phosphoadenylate-(3´-5´)-adenosine (pApA) and/or two molecules of adenosine monophosphate (AMP) (Commichau et al. 2019).

To date, five distinct types of DACs have been characterized: DisA, CdaA, CdaS, CdaM, and CdaZ (Commichau et al. 2019). DisA, containing an N-terminal DAC domain and a C-terminal DNA-binding Helix-hairpin-Helix domain, is the sole DAC domain protein in Actinobacteria (Witte et al. 2008, Latoscha et al. 2019). In *S. venezuelae*, DisA remains at constant levels throughout the developmental cycle and represents the only source for c-di-AMP during the transition to sporulation and the sporulation phase. However, during early developmental growth, low levels of c-di-AMP were detectable even in the *disA* mutant, suggesting the presence of another non-DAC domain c-di-AMP producing enzyme in this species (Latoscha et al. 2020). As in many other bacterial models, c-di-AMP plays an important role in osmostress response in *Streptomyces* and contributes to resistance towards upshifts of ionic osmolytes. *S. venezuelae* ∆*disA* shows a severe growth defect on nutrient agar containing either 0.5 M NaCl or 0.5 M KCl but grows and develops comparable to the wildtype strain in the presence of 0.5 M sucrose and on standard sporulation agar (Latoscha et al. 2020) (Andreas Latoscha and Natalia Tschowri, unpublished data).

**Figure 3:**
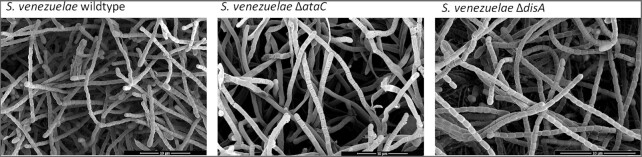
Deletion of *ataC* compromises *S. venezuelae* development. Scanning electron micrographs showing that after 4 days of incubation on MYM agar, *S. venezuelae* wildtype (left) and Δ*disA* (right) formed spores. In comparison, the *ataC* mutant (centre) formed predominantly non-sporulating aerial hyphae and had many flat, likely lysed hyphae.

To stop c-di-AMP signals, the molecule is hydrolyzed by specific DHH-DHHA1 and HD-domains (Rao et al. 2010, Huynh et al. 2015). It was long unsolved how the majority of Actinobacteria terminate c-di-AMP signals since they possess DisA for c-di-AMP production but many of them lack PDEs of the DHH-DHHA1 and HD-type. We found that they use a new type of c-di-AMP PDEs that we named AtaC for actinobacterial PDE targeting c-di-AMP. AtaC degrades c-di-AMP to AMP via pApA and belongs to the type I PDE/nucleotide pyrophosphatase family of proteins. It is highly conserved in Actinobacteria, including a number of species of the genus *Mycobacteria* (Latoscha et al. 2020, Yin et al. 2020). Deletion of *ataC* in *S. venezuelae* leads to a 1.5–2-fold increase of c-di-AMP levels, which compromises growth in liquid MYM and on agar plates. In addition, inactivation of the active site residue D_269_ in AtaC or deletion of the gene causes a severe sporulation defect and a grey colony morphology, suggesting that development and the formation of the green spore pigment are affected by elevated c-di-AMP (Latoscha et al. 2020). Notably, addition of osmolytes to agar medium has no added effect on the growth of *S. venezuelae* ∆*ataC*. However, as judged by transmission electron micrographs of the *ataC* mutant, many of the aerial hyphae formed by the strain appear to have shrinked or lysed. One possible explanation for this phenotype is that high c-di-AMP levels in ∆*ataC* cause loss of turgor even in the absence of osmotic stress challenges (Fig. 3).

**Figure 4:**
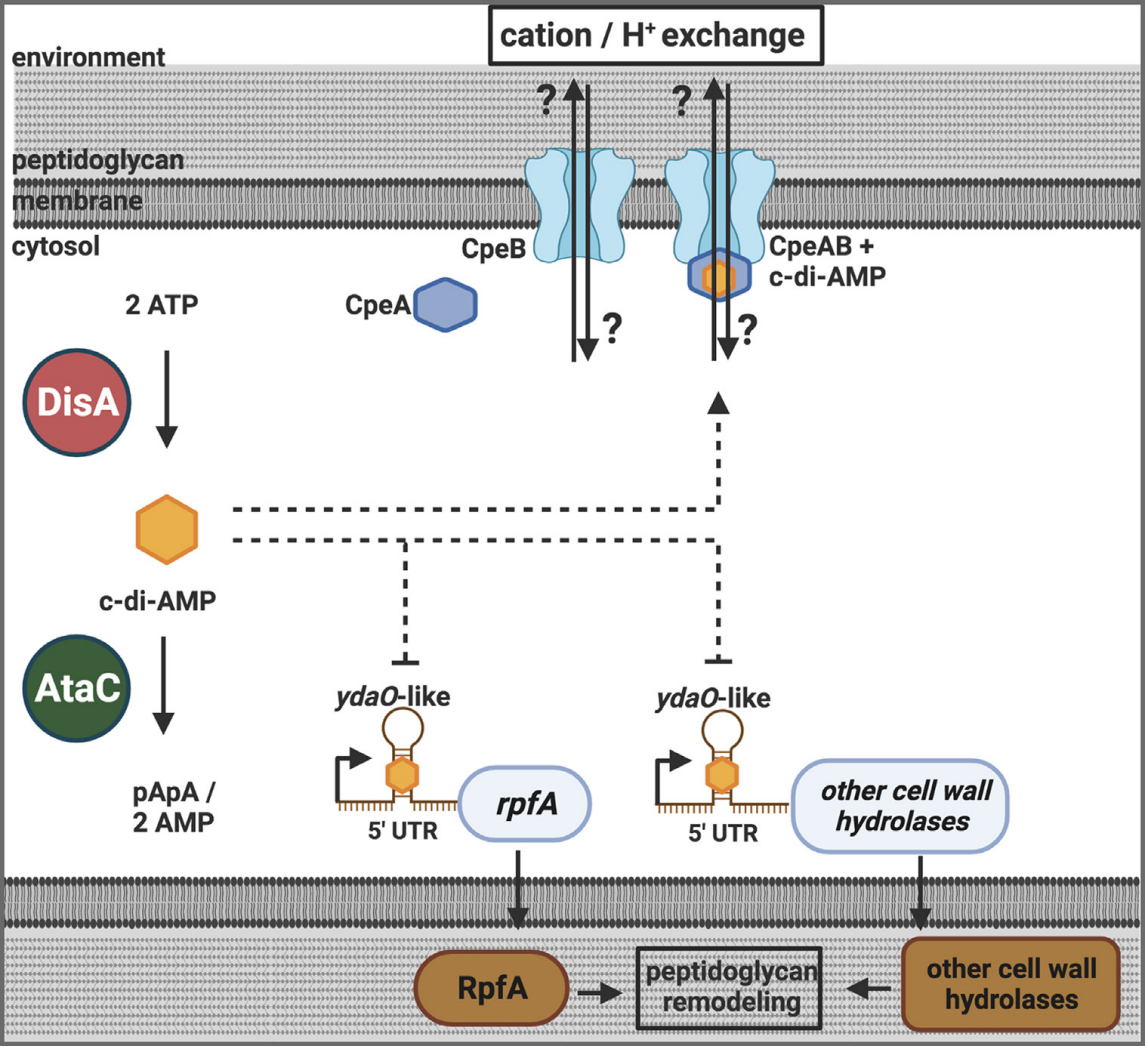
c-di-AMP metabolism and signalling in *Streptomyces*. The DAC-domain protein DisA produces c-di-AMP out of two ATP molecules. The PDE AtaC hydrolyzes c-di-AMP to two AMPs via 5´-pApA. C-di-AMP binds to the RCK_C domain of CpeA and stimulates interaction with CpeB. The CpeAB system likely represents a KhtU-like K^+^/H^+^ antiporter, as supported by homology analysis (Latoscha et al. 2020). Additionally, c-di-AMP binds to the *ydaO*-like riboswitch in the 5´-UTR of *rpfA*, leading to transcription termination of *rpfA* (St-Onge and Elliot 2017). RpfA encodes a cell wall hydrolase involved in cell wall remodelling during germination and growth. In *S. coelicolor*, the *ydaO*-like riboswitch has also been found in the 5´-UTR of six other genes encoding putative cell wall hydrolases (Haiser et al. 2009).

It is still puzzling why depletion and elevation of c-di-AMP by deletion of *disA* and inactivation of AtaC, respectively, do not have contrary effects but rather seem to cause disconnected phenotypes. While the *disA* mutant grows and develops normally under standard conditions but is highly susceptible to ionic osmostress, the *ataC* mutant has a general growth and developmental defect that does not seem to be further affected by the addition of osmolytes (Latoscha et al. 2020). The key to our understanding how c-di-AMP controls *Streptomyces* physiology is the identification of c-di-AMP effector molecules. To date, two types of c-di-AMP effectors have been found in the genus: the *ydaO*-like riboswitch and the RCK_C domain (St-Onge et al. 2015, Latoscha et al. 2020). Riboswitches are cis-acting RNAs mostly located in the 5´-untranslated region (UTR) of the target mRNA. The regulatory element contains two domains: an upstream sensor domain that binds to the specific ligand and the expression platform that regulates the downstream coding sequences by modulating transcript elongation, transcript stability, or translation initiation (Serganov and Nudler 2013). The *ydaO* riboswitch is present in the UTRs of mRNAs that specify potassium and amino acid transporters and enzymes for cell wall homeostasis (Nelson et al. 2013). *S. coelicolor* and *S. venezuelae* encode seven and six cell wall hydrolases, respectively, containing an *ydaO*-like riboswitch in their 5´-UTR (Haiser et al. 2009, Latoscha et al. 2019). Binding of c-di-AMP to the *ydaO*-like riboswitch and its consequences have been reported only for *rpfA* (St-Onge et al. 2015, St-Onge and Elliot 2017). *rpfA* codes for a protein belonging to a class of ‘resuscitation-promoting factors’ (Rpfs) that have lysozyme-like enzymatic activity, thereby cleaving the sugar backbone of peptidoglycan and promote resuscitation of the dormant cells (Telkov et al. 2006). *S. coelicolor and S. venezuelae* share extensive sequence and structural similarity in the 5´-UTRs of *rpfA*. In *S. venezuelae, rpfA* transcripts were increased by five-fold in the *disA* mutant, and binding of c-di-AMP to the 5´-UTR of *S. coelicolor rpfA* was shown *in vitro*. Upon binding to c-di-AMP, the riboswitch undergoes a conformational change, resulting in premature *rpfA* transcription termination (St-Onge and Elliot 2017). In *S. coelicolor*, both overexpression and deletion of *rpfA* delay spore germination, allowing the conclusion that via the *ydaO*-like riboswitch, c-di-AMP regulates transcription of *rpfA* and probably of other cell-wall hydrolases to establish timely germination and outgrowth of spores (St-Onge et al. 2015) (Fig. 4).

In Firmicutes, RCK_C domains are involved in the control of potassium homeostasis by modulating the activity of a number of potassium transporters (see above) (Stülke and Krüger 2020). RCK_C domains are established direct targets of c-di-AMP that have the (I/L) (I/L)X_2_DX_1_RX_5_N(I/L) (I/L) signature for dinucleotide binding (Schrecker et al. 2019). Our *in silico* analysis revealed that *S. venezuelae* has six RCK_C domain-containing proteins: Vnz_28055, Vnz_28520, Vnz_28040, Vnz_14905, Vnz_27460, and Vnz_12665. We have purified the six RCK_C domains and found that CpeA and Vnz_28520 bind c-di-AMP, while others do not (Latoscha et al. 2020) (Andreas Latoscha and Natalia Tschowri, unpublished data). This is in line with our finding that the c-di-AMP binding site is most conserved in CpeA and Vnz_28520. *cpeA* forms a conserved operon with *cpeB* (*vnz_28050*), encoding a protein with 13 predicted transmembrane helices (Table 1). In addition, *S. venezuelae* has a small open reading frame in the same operon, *cpeC* (*vnz_28045*) (Latoscha et al. 2020). Based on our sequence analysis, the Cpe system appears to be homologous to the KhtTUS K^+^/H^+^ antiporter from *B. subtilis* (Cereija et al. 2021). CpeA shares 27% identical residues with KhtT, 32% of amino acids are identical between KhtU and CpeB, and, finally, CpeC is to 22% identical with KhtS. Of note, the second c-di-AMP-binding RCK_C domain protein, Vnz_28520, forms an operon with the predicted transporter Vnz_28525. Based on our sequence alignment, the two genes seem to be a duplication of the Cpe system, which lacks CpeC and is not well conserved in streptomycetes. c-di-AMP activates KhtTU-mediated K^+^ export in *B. subtilis* by a mechanism that has been elucidated in detail in the study by Cereija et al. (2021). The authors demonstrate that one c-di-AMP binds at the KhtT dimer interface, which disrupts the KhtT-KhtU interaction, leading to full activation of the KhtU-mediated K^+^/H^+^ antiport. Interestingly, KhtTU activation by c-di-AMP is highly dependent on pH and increases about 60-fold when the pH was raised from 7.5 to 8.5. The authors propose that at alkaline conditions, H^+^ import mediated by KhtU becomes critical for pH homeostasis (Cereija et al. 2021). Considering that the Cpe system from *S. venezuelae* shows pronounced homology to the Kht system from *B. subtilis* and CpeB belongs to the cation/proton exchanger family (Table 1), it is likely that CpeB also acts as a K^+^ export/H^+^ import system. However, the consequences of c-di-AMP interaction with CpeA in *Streptomyces* or with KhtT in *Bacilli*, respectively, seem to differ. While c-di-AMP weakens complex formation between KhtT and KhtU, it stimulates interaction between CpeA and CpeB, as shown using bacterial adenylate cyclase two-hybrid assays that were performed in the presence of either an active or inactive DisA in *E. coli* (Latoscha et al. 2020). Thus, it remains to be clarified whether c-di-AMP activates or inhibits CpeB function and whether the system is indeed a K^+^/H^+^ antiporter (Fig. 4).

## Conclusions

In response to osmotic upshifts, filamentous *Streptomyces* arrest their growth from the main hyphal tip and form multiple new branches from the lateral sides. This stress phenotype raises multiple questions such as why rearrangement of cell polarity is beneficial for survival of osmotic challenges and which signalling and regulatory cascades underlie the complex reprogramming of apical growth. In addition, high salinity conditions block spore formation, presumably allowing the cell to invest the needed energy into stress rescue processes instead of the elaborate sporulation programme that may not be successfully completed. Block of cell differentiation is likely mediated by the induced expression and activity of alternative sigma factors, such as σ^B^, which alter the transcriptional profile of the cell, including the expression of genes encoding the osmoresponsive response regulator OsaB and developmental regulators. As in other classical models, the accumulation of potassium is the first emergency response of *Streptomyces* to osmotic upshifts. But, we know very little about osmolyte transport systems that are central to *Streptomyces* adaptation to osmotic stress. The only biochemically well studied K^+^ uptake system in the genus *Streptomyces* is the potassium channel KcsA, which is, however, not well conserved, and its physiological role and contribution to osmoadaptation have not been fully resolved yet. Similarly, although it is well described that *Streptomyces* use free amino acids (mainly proline), ectoines, and trehalose as compatible solutes, uptake systems for any osmoprotectant have not been characterized yet. Clearly, c-di-AMP is important for survival at high salinity conditions, but how the second messenger improves survival at such stress conditions is not completely understood yet and requires the identification of additional c-di-AMP effectors and a better mechanistic understanding of the known c-di-AMP targets, CpeA and the *ydaO*-like riboswitch-controlled cell wall hydrolases. In summary, identification and understanding of cellular components and processes that enable streptomycetes to cope with osmotic stress are not only fundamental for their performance as cell factories in biotechnology but also for their ecophysiology in their natural habitat and as a unique model for filamentous, multicellular bacteria with a complex developmental life cycle.

## References

[bib1] Akai M , Onai K, Morishita M et al. Aquaporin AqpZ is involved in cell volume regulation and sensitivity to osmotic stress in *Synechocystis* sp. strain PCC 6803. J Bacteriol. 2012;194:6828–36. 23043001 10.1128/JB.01665-12 PMC3510584

[bib2] Asencion Diez MD , Miah F, Stevenson CE et al. The production and utilization of GDP-glucose in the biosynthesis of trehalose 6-phosphate by *Streptomyces venezuelae*. J Biol Chem. 2017;292:945–54. 27903647 10.1074/jbc.M116.758664 PMC5247666

[bib3] Bibb MJ . Regulation of secondary metabolism in streptomycetes. Curr Opin Microbiol. 2005;8:208–15. 15802254 10.1016/j.mib.2005.02.016

[bib4] Bibb MJ , Domonkos A, Chandra G et al. Expression of the chaplin and rodlin hydrophobic sheath proteins in *Streptomyces venezuelae* is controlled by sigma(BldN) and a cognate anti-sigma factor. Mol Microbiol. 2012;84:1033–49. 22582857 10.1111/j.1365-2958.2012.08070.x

[bib5] Bishop A , Fielding S, Dyson P et al. Systematic insertional mutagenesis of a streptomycete genome: a link between osmoadaptation and antibiotic production. Genome Res. 2004;14:893–900. 15078860 10.1101/gr.1710304 PMC479117

[bib6] Booth IR . Bacterial mechanosensitive channels: progress towards an understanding of their roles in cell physiology. Curr Opin Microbiol. 2014;18:16–22. 24607989 10.1016/j.mib.2014.01.005 PMC4005912

[bib7] Bramkamp M , Altendorf K, Greie JC. Common patterns and unique features of P-type ATPases: a comparative view on the KdpFABC complex from *Escherichia coli*. Mol Membr Biol. 2007;24:375–86. 17710642 10.1080/09687680701418931

[bib8] Braña AF , Mendez C, Diaz LA et al. Glycogen and trehalose accumulation during colony development in *Streptomyces antibioticus*. J Gen Microbiol. 1986;132:1319–26. 3534138 10.1099/00221287-132-5-1319

[bib9] Bremer E , Krämer R. Responses of microorganisms to osmotic stress. Annu Rev Microbiol. 2019;73:313–34. 31180805 10.1146/annurev-micro-020518-115504

[bib10] Buda R , Liu Y, Yang J et al. Dynamics of *Escherichia coli’s* passive response to a sudden decrease in external osmolarity. Proc Nat Acad Sci USA. 2016;113:E5838–E46. 27647888 10.1073/pnas.1522185113 PMC5056102

[bib11] Bursy J , Kuhlmann AU, Pittelkow M et al. Synthesis and uptake of the compatible solutes ectoine and 5-hydroxyectoine by *Streptomyces coelicolor* A3(2) in response to salt and heat stresses. Appl Environ Microbiol. 2008;74:7286–96. 18849444 10.1128/AEM.00768-08 PMC2592907

[bib12] Bush MJ , Tschowri N, Schlimpert S et al. c-di-GMP signalling and the regulation of developmental transitions in streptomycetes. Nat Rev Microbiol. 2015;13:749–60. 26499894 10.1038/nrmicro3546

[bib13] Cereija TB , Guerra JPL, Jorge JMP et al. c-di-AMP, a likely master regulator of bacterial K(+) homeostasis machinery, activates a K(+) exporter. Proc Natl Acad Sci. 2021;118:e2020653118. 33790011 10.1073/pnas.2020653118 PMC8040634

[bib14] Chater KF , Biro S, Lee KJ et al. The complex extracellular biology of *Streptomyces*. FEMS Microbiol Rev. 2010;34:171–98. 20088961 10.1111/j.1574-6976.2009.00206.x

[bib15] Claessen D , Rink R, de JW et al. A novel class of secreted hydrophobic proteins is involved in aerial hyphae formation in *Streptomyces coelicolor* by forming amyloid-like fibrils. Genes Dev. 2003;17:1714–26. 12832396 10.1101/gad.264303 PMC196180

[bib16] Commichau FM , Heidemann JL, Ficner R et al. Making and breaking of an essential poison: the cyclases and phosphodiesterases that produce and degrade the essential second messenger cyclic di-AMP in bacteria. J Bacteriol. 2019;201:e00462–18. 30224435 10.1128/JB.00462-18 PMC6287462

[bib17] Corrigan RM , Grundling A Cyclic di-AMP: another second messenger enters the fray. Nat Rev Microbiol. 2013;11:513–24. 23812326 10.1038/nrmicro3069

[bib18] Czech L , Hermann L, Stoveken N et al. Role of the extremolytes ectoine and hydroxyectoine as stress protectants and nutrients: genetics, phylogenomics, biochemistry, and structural analysis. Genes. 2018;9:177. 29565833 10.3390/genes9040177 PMC5924519

[bib19] de Jong W , Vijgenboom E, Dijkhuizen L et al. SapB and the rodlins are required for development of *Streptomyces coelicolor* in high osmolarity media. FEMS Microbiol Lett. 2012;329:154–9. 22309453 10.1111/j.1574-6968.2012.02517.x

[bib20] Delamarche C , Thomas D, Rolland JP et al. Visualization of AqpZ-mediated water permeability in *Escherichia coli* by cryoelectron microscopy. J Bacteriol. 1999;181:4193–7. 10400575 10.1128/jb.181.14.4193-4197.1999 PMC93919

[bib21] Doyle DA , Morais Cabral J, Pfuetzner RA et al. The structure of the potassium channel: molecular basis of K+ conduction and selectivity. Science. 1998;280:69–77. 9525859 10.1126/science.280.5360.69

[bib22] Edwards MD , Black S, Rasmussen T et al. Characterization of three novel mechanosensitive channel activities in *Escherichia coli*. Channels. 2012;6:272–81. 22874652 10.4161/chan.20998 PMC3508906

[bib23] Elbein AD , Pan YT, Pastuszak I et al. New insights on trehalose: a multifunctional molecule. Glycobiology. 2003;13:17R–27R. 10.1093/glycob/cwg047 12626396

[bib24] Elbourne LD , Tetu SG, Hassan KA et al. TransportDB 2.0: a database for exploring membrane transporters in sequenced genomes from all domains of life. Nucleic Acids Res. 2017;45:D320–4. 27899676 10.1093/nar/gkw1068 PMC5210551

[bib25] Elliot MA , Karoonuthaisiri N, Huang J et al. The chaplins: a family of hydrophobic cell-surface proteins involved in aerial mycelium formation in *Streptomyces coelicolor*. Genes Dev. 2003;17:1727–40. 12832397 10.1101/gad.264403 PMC196181

[bib26] Empadinhas N , da Costa MS. Osmoadaptation mechanisms in prokaryotes: distribution of compatible solutes. Int Microbiol. 2008;11:151–61. 18843593

[bib27] Fernandez Martinez L , Bishop A, Parkes L et al. Osmoregulation in *Streptomyces coelicolor*: modulation of SigB activity by OsaC. Mol Microbiol. 2009;71:1250–62. 19154327 10.1111/j.1365-2958.2009.06599.x

[bib28] Fernandez-Moreno MA , Caballero JL, Hopwood DA et al. The act cluster contains regulatory and antibiotic export genes, direct targets for translational control by the *bldA* tRNA gene of *Streptomyces*. Cell. 1991;66:769–80. 1878971 10.1016/0092-8674(91)90120-n

[bib29] Flärdh K . Essential role of DivIVA in polar growth and morphogenesis in *Streptomyces coelicolor* A3(2). Mol Microbiol. 2003;49:1523–36. 12950918 10.1046/j.1365-2958.2003.03660.x

[bib30] Flärdh K , Buttner MJ. *Streptomyces* morphogenetics: dissecting differentiation in a filamentous bacterium. Nat Rev Microbiol. 2009;7:36–49. 19079351 10.1038/nrmicro1968

[bib31] Flärdh K , Richards DM, Hempel AM et al. Regulation of apical growth and hyphal branching in *Streptomyces*. Curr Opin Microbiol. 2012;15:737–43. 23153774 10.1016/j.mib.2012.10.012

[bib32] Frojd MJ , Flärdh K. Extrusion of extracellular membrane vesicles from hyphal tips of *Streptomyces venezuelae* coupled to cell-wall stress. Microbiology. 2019;165:1295–305. 31282851 10.1099/mic.0.000836

[bib33] Frojd MJ , Flärdh K. Apical assemblies of intermediate filament-like protein FilP are highly dynamic and affect polar growth determinant DivIVA in *Streptomyces venezuelae*. Mol Microbiol. 2019;112:47–61. 30929261 10.1111/mmi.14253

[bib34] Fuchino K , Flärdh K, Dyson P et al. Cell-biological studies of osmotic shock response in *Streptomyces* spp. J Bacteriol. 2017;199:e00465–16. 27795320 10.1128/JB.00465-16 PMC5165099

[bib35] Galinski EA , Pfeiffer HP, Truper HG. 1,4,5,6-Tetrahydro-2-methyl-4-pyrimidinecarboxylic acid. A novel cyclic amino acid from halophilic phototrophic bacteria of the genus *Ectothiorhodospira*. Eur J Biochem. 1985;149:135–9. 3838936 10.1111/j.1432-1033.1985.tb08903.x

[bib36] Godinez O , Dyson P, del Sol R et al. Targeting the osmotic stress response for strain improvement of an industrial producer of secondary metabolites. J Microbiol Biotechnol. 2015;25:1787–95. 26139611 10.4014/jmb.1503.03042

[bib37] Gundlach J , Kruger L, Herzberg C et al. Sustained sensing in potassium homeostasis: cyclic di-AMP controls potassium uptake by KimA at the levels of expression and activity. J Biol Chem. 2019;294:9605–14. 31061098 10.1074/jbc.RA119.008774 PMC6579464

[bib38] Haiser HJ , Yousef MR, Elliot MA. Cell wall hydrolases affect germination, vegetative growth, and sporulation in *Streptomyces coelicolor*. J Bacteriol. 2009;191:6501–12. 19717604 10.1128/JB.00767-09 PMC2795291

[bib39] Hempel AM , Cantlay S, Molle V et al. The Ser/Thr protein kinase AfsK regulates polar growth and hyphal branching in the filamentous bacteria *Streptomyces*. Proc Nat Acad Sci USA. 2012;109:E2371–9. 22869733 10.1073/pnas.1207409109 PMC3435184

[bib40] Hermann L , Mais CN, Czech L et al. The ups and downs of ectoine: structural enzymology of a major microbial stress protectant and versatile nutrient. Biol Chem. 2020;401:1443–68. 32755967 10.1515/hsz-2020-0223

[bib41] Hey-Ferguson A , Mitchell M, Elbein AD. Trehalose metabolism in germinating spores of *Streptomyces hygroscopicus*. J Bacteriol. 1973;116:1084–5. 4355488 10.1128/jb.116.2.1084-1085.1973 PMC285504

[bib42] Hirano M , Onishi Y, Yanagida T et al. Role of the KcsA channel cytoplasmic domain in pH-dependent gating. Biophys J. 2011;101:2157–62. 22067153 10.1016/j.bpj.2011.09.024 PMC3207171

[bib43] Hoffmann T , Bremer E. Guardians in a stressful world: the Opu family of compatible solute transporters from *Bacillus subtilis*. Biol Chem. 2017;398:193–214. 27935846 10.1515/hsz-2016-0265

[bib44] Holmes NA , Walshaw J, Leggett RM et al. Coiled-coil protein Scy is a key component of a multiprotein assembly controlling polarized growth in *Streptomyces*. Proc Nat Acad Sci USA. 2013;110:E397–406. 23297235 10.1073/pnas.1210657110 PMC3562780

[bib45] Hopwood DA . Streptomyces in Nature and Medicine: The Antibiotic Makers. New York: Oxford University Press, 2007.

[bib46] Huang CS , Pedersen BP, Stokes DL. Crystal structure of the potassium-importing KdpFABC membrane complex. Nature. 2017;546:681–5. 28636601 10.1038/nature22970 PMC5495170

[bib47] Huynh TN , Luo S, Pensinger D et al. An HD-domain phosphodiesterase mediates cooperative hydrolysis of c-di-AMP to affect bacterial growth and virulence. Proc Nat Acad Sci USA. 2015;112:E747–56. 25583510 10.1073/pnas.1416485112 PMC4343097

[bib48] Inbar L , Lapidot A. The structure and biosynthesis of new tetrahydropyrimidine derivatives in actinomycin D producer *Streptomyces parvulus*. Use of 13C- and 15N-labeled L-glutamate and 13C and 15 N NMR spectroscopy. J Biol Chem. 1988;263:16014–22. 2903148

[bib49] Iturriaga G , Suarez R, Nova-Franco B. Trehalose metabolism: from osmoprotection to signaling. Int J Mol Sci. 2009;10:3793–810. 19865519 10.3390/ijms10093793 PMC2769160

[bib50] Jakimowicz D , van Wezel GP. Cell division and DNA segregation in *Streptomyces*: how to build a septum in the middle of nowhere?. Mol Microbiol. 2012;85:393–404. 22646484 10.1111/j.1365-2958.2012.08107.x

[bib51] Jiang Y , Lee A, Chen J et al. Crystal structure and mechanism of a calcium-gated potassium channel. Nature. 2002;417:515–22. 12037559 10.1038/417515a

[bib52] Kelemen GH , Brian P, Flärdh K et al. Developmental regulation of transcription of *whiE*, a locus specifying the polyketide spore pigment in *Streptomyces coelicolor* A3 (2). J Bacteriol. 1998;180:2515–21. 9573206 10.1128/jb.180.9.2515-2521.1998 PMC107196

[bib53] Killham K , Firestone MK. Salt stress control of intracellular solutes in streptomycetes indigenous to saline soils. Appl Environ Microbiol. 1984;47:301–6. 16346472 10.1128/aem.47.2.301-306.1984 PMC239664

[bib54] Kodani S , Hudson ME, Durrant MC et al. The SapB morphogen is a lantibiotic-like peptide derived from the product of the developmental gene *ramS* in *Streptomyces coelicolor*. Proc Natl Acad Sci. 2004;101:11448–53. 15277670 10.1073/pnas.0404220101 PMC509221

[bib55] Kol S , Merlo ME, Scheltema RA et al. Metabolomic characterization of the salt stress response in *Streptomyces coelicolor*. Appl Environ Microbiol. 2010;76:2574–81. 20190082 10.1128/AEM.01992-09 PMC2849216

[bib56] Krämer R . Osmosensing and osmosignaling in *Corynebacterium glutamicum*. Amino Acids. 2009;37:487–97. 19308662 10.1007/s00726-009-0271-6

[bib57] Laermann V , Cudic E, Kipschull K et al. The sensor kinase KdpD of *Escherichia coli* senses external K+. Mol Microbiol. 2013;88:1194–204. 23651428 10.1111/mmi.12251

[bib58] Latoscha A , Drexler DJ, Al-Bassam MM et al. c-di-AMP hydrolysis by the phosphodiesterase AtaC promotes differentiation of multicellular bacteria. Proc Natl Acad Sci. 2020;117:7392–400. 32188788 10.1073/pnas.1917080117 PMC7132281

[bib59] Latoscha A , Wormann ME, Tschowri N. Nucleotide second messengers in *Streptomyces*. Microbiology. 2019;165:1153–65. 31535967 10.1099/mic.0.000846

[bib60] Leaver M , Dominguez-Cuevas P, Coxhead JM et al. Life without a wall or division machine in *Bacillus subtilis*. Nature. 2009;457:849–53. 19212404 10.1038/nature07742

[bib61] Lee EJ , Karoonuthaisiri N, Kim HS et al. A master regulator sigmaB governs osmotic and oxidative response as well as differentiation via a network of sigma factors in *Streptomyces coelicolor*. Mol Microbiol. 2005;57:1252–64. 16101999 10.1111/j.1365-2958.2005.04761.x

[bib62] Leonardo MR , Forst S. Re-examination of the role of the periplasmic domain of EnvZ in sensing of osmolarity signals in *Escherichia coli*. Mol Microbiol. 1996;22:405–13. 8939425 10.1046/j.1365-2958.1996.1271487.x

[bib63] Liu G , Chater KF, Chandra G et al. Molecular regulation of antibiotic biosynthesis in *Streptomyces*. Microbiol Mol Biol Rev. 2013;77:112–43. 23471619 10.1128/MMBR.00054-12 PMC3591988

[bib64] McBride MJ , Ensign JC. Regulation of trehalose metabolism by *Streptomyces griseus* spores. J Bacteriol. 1990;172:3637–43. 2113908 10.1128/jb.172.7.3637-3643.1990 PMC213337

[bib65] McLean TC , Lo R, Tschowri N et al. Sensing and responding to diverse extracellular signals: an updated analysis of the sensor kinases and response regulators of *Streptomyces* species. Microbiology. 2019;165:929–52. 31334697 10.1099/mic.0.000817

[bib66] Moker N , Brocker M, Schaffer S et al. Deletion of the genes encoding the MtrA-MtrB two-component system of *Corynebacterium glutamicum* has a strong influence on cell morphology, antibiotics susceptibility and expression of genes involved in osmoprotection. Mol Microbiol. 2004;54:420–38. 15469514 10.1111/j.1365-2958.2004.04249.x

[bib67] Nelson JW , Sudarsan N, Furukawa K et al. Riboswitches in eubacteria sense the second messenger c-di-AMP. Nat Chem Biol. 2013;9:834–9. 24141192 10.1038/nchembio.1363 PMC3830699

[bib68] Passot FM , Cantlay S, Flardh K. Protein phosphatase SppA regulates apical growth and dephosphorylates cell polarity determinant DivIVA in *Streptomyces coelicolor*. Mol Microbiol. 2022;117:411–28. 34862689 10.1111/mmi.14856

[bib69] Pastor JM , Salvador M, Argandona M et al. Ectoines in cell stress protection: uses and biotechnological production. Biotechnol Adv. 2010;28:782–801. 20600783 10.1016/j.biotechadv.2010.06.005

[bib70] Ramijan K , Ultee E, Willemse J et al. Stress-induced formation of cell wall-deficient cells in filamentous actinomycetes. Nat Commun. 2018;9:5164. 30514921 10.1038/s41467-018-07560-9 PMC6279842

[bib71] Rao F , See RY, Zhang D et al. YybT is a signaling protein that contains a cyclic dinucleotide phosphodiesterase domain and a GGDEF domain with ATPase activity. J Biol Chem. 2010;285:473–82. 19901023 10.1074/jbc.M109.040238 PMC2804195

[bib72] Richards DM , Hempel AM, Flardh K et al. Mechanistic basis of branch-site selection in filamentous bacteria. PLoS Comput Biol. 2012;8:e1002423. 22423220 10.1371/journal.pcbi.1002423 PMC3297577

[bib73] Rudd BA , Hopwood DA. A pigmented mycelial antibiotic in *Streptomyces coelicolor*: control by a chromosomal gene cluster. J Gen Microbiol. 1980;119:333–40. 7229612 10.1099/00221287-119-2-333

[bib74] Rueda B , Miguelez EM, Hardisson C et al. Changes in glycogen and trehalose content of *Streptomyces brasiliensis* hyphae during growth in liquid cultures under sporulating and non-sporulating conditions. FEMS Microbiol Lett. 2001;194:181–5. 11164305 10.1111/j.1574-6968.2001.tb09466.x

[bib75] Schneider D , Bruton CJ, Chater KF. Duplicated gene clusters suggest an interplay of glycogen and trehalose metabolism during sequential stages of aerial mycelium development in *Streptomyces coelicolor* A3(2). Molecular and General Genetics MGG. 2000;263:543–53. 10821190 10.1007/s004380051200

[bib76] Schrecker M , Wunnicke D, Hanelt I. How RCK domains regulate gating of K+ channels. Biol Chem. 2019;400:1303–22. 31361596 10.1515/hsz-2019-0153

[bib77] Schrempf H , Schmidt O, Kummerlen R et al. A prokaryotic potassium ion channel with two predicted transmembrane segments from *Streptomyces lividans*. EMBO J. 1995;14:5170–8. 7489706 10.1002/j.1460-2075.1995.tb00201.x PMC394625

[bib78] Schumacher MA , Wormann ME, Henderson M et al. Allosteric regulation of glycogen breakdown by the second messenger cyclic di-GMP. Nat Commun. 2022;13:5834. 36192422 10.1038/s41467-022-33537-w PMC9530166

[bib79] Serganov A , Nudler E. A decade of riboswitches. Cell. 2013;152:17–24. 23332744 10.1016/j.cell.2012.12.024 PMC4215550

[bib80] Sevcikova B , Kormanec J. Differential production of two antibiotics of *Streptomyces coelicolor* A3(2), actinorhodin and undecylprodigiosin, upon salt stress conditions. Arch Microbiol. 2004;181:384–9. 15054568 10.1007/s00203-004-0669-1

[bib81] Sleator RD , Hill C. Bacterial osmoadaptation: the role of osmolytes in bacterial stress and virulence. FEMS Microbiol Rev. 2002;26:49–71. 12007642 10.1111/j.1574-6976.2002.tb00598.x

[bib82] Som NF , Heine D, Holmes N et al. The MtrAB two-component system controls antibiotic production in *Streptomyces coelicolor* A3(2). Microbiology. 2017;163:1415–9. 28884676 10.1099/mic.0.000524 PMC5845573

[bib83] Som NF , Heine D, Holmes NA et al. The conserved actinobacterial two-component system MtrAB coordinates chloramphenicol production with sporulation in *Streptomyces venezuelae* NRRL B-65442. Front Microbiol. 2017b;8:1145. 28702006 10.3389/fmicb.2017.01145 PMC5487470

[bib84] Spasic J , Mandic M, Djokic L et al. *Streptomyces* spp. in the biocatalysis toolbox. Appl Microbiol Biotechnol. 2018;102:3513–36. 29502181 10.1007/s00253-018-8884-x

[bib85] Stautz J , Hellmich Y, Fuss MF et al. Molecular mechanisms for bacterial potassium homeostasis. J Mol Biol. 2021;433:166968. 33798529 10.1016/j.jmb.2021.166968 PMC9041122

[bib86] Stock AM , Robinson VL, Goudreau PN. Two-component signal transduction. Annu Rev Biochem. 2000;69:183–215. 10966457 10.1146/annurev.biochem.69.1.183

[bib87] St-Onge RJ , Elliot MA. Regulation of a muralytic enzyme-encoding gene by two non-coding RNAs. RNA Biol. 2017;14:1592–605. 28640671 10.1080/15476286.2017.1338241 PMC5785216

[bib88] St-Onge RJ , Haiser HJ, Yousef MR et al. Nucleotide second messenger-mediated regulation of a muralytic enzyme in *Streptomyces*. Mol Microbiol. 2015;96:779–95. 25682701 10.1111/mmi.12971

[bib89] Strom AR , Kaasen I. Trehalose metabolism in *Escherichia coli*: stress protection and stress regulation of gene expression. Mol Microbiol. 1993;8:205–10. 8391102 10.1111/j.1365-2958.1993.tb01564.x

[bib90] Stülke J , Krüger L. Cyclic di-AMP signaling in bacteria. Annu Rev Microbiol. 2020;74:159–79. 32603625 10.1146/annurev-micro-020518-115943

[bib91] Tanghe A , Van Dijck P, Thevelein JM. Why do microorganisms have aquaporins?. Trends Microbiol. 2006;14:78–85. 16406529 10.1016/j.tim.2005.12.001

[bib92] Tascon I , Sousa JS, Corey RA et al. Structural basis of proton-coupled potassium transport in the KUP family. Nat Commun. 2020;11:626. 32005818 10.1038/s41467-020-14441-7 PMC6994465

[bib93] Telkov MV , Demina GR, Voloshin SA et al. Proteins of the Rpf (resuscitation promoting factor) family are peptidoglycan hydrolases. Biochemistry (Mosc). 2006;71:414–22. 16615861 10.1134/s0006297906040092

[bib94] van Bergeijk DA , Terlouw BR, Medema MH et al. Ecology and genomics of Actinobacteria: new concepts for natural product discovery. Nat Rev Microbiol. 2020;18:546–58. 32483324 10.1038/s41579-020-0379-y

[bib95] Viollier PH , Kelemen GH, Dale GE et al. Specialized osmotic stress response systems involve multiple SigB-like sigma factors in *Streptomyces coelicolor*. Mol Microbiol. 2003;47:699–714. 12535070 10.1046/j.1365-2958.2003.03302.x

[bib96] Wang CX , Ge HX, Hou XP et al. Roles of larger conductance mechanosensitive channels (MscL) in sporulation and Act secretion in *Streptomyces coelicolor*. J Basic Microbiol. 2007;47:518–24. 18072238 10.1002/jobm.200700238

[bib97] Whatmore AM , Chudek JA, Reed RH. The effects of osmotic upshock on the intracellular solute pools of *Bacillus subtilis*. J Gen Microbiol. 1990;136:2527–35. 2127802 10.1099/00221287-136-12-2527

[bib98] Widderich N , Rodrigues CD, Commichau FM et al. Salt-sensitivity of sigma(H) and Spo0A prevents sporulation of *Bacillus subtilis* at high osmolarity avoiding death during cellular differentiation. Mol Microbiol. 2016;100:108–24. 26712348 10.1111/mmi.13304 PMC4992981

[bib99] Willey J , Santamaria R, Guijarro J et al. Extracellular complementation of a developmental mutation implicates a small sporulation protein in aerial mycelium formation by *S. coelicolor*. Cell. 1991;65:641–50. 2032288 10.1016/0092-8674(91)90096-h

[bib100] Witte G , Hartung S, Buttner K et al. Structural biochemistry of a bacterial checkpoint protein reveals diadenylate cyclase activity regulated by DNA recombination intermediates. Mol Cell. 2008;30:167–78. 18439896 10.1016/j.molcel.2008.02.020

[bib101] Wood JM . Osmosensing by bacteria: signals and membrane-based sensors. Microbiology and molecular biology reviews: Microbiol Mol Biol Rev. 1999;63:230–62. 10066837 10.1128/mmbr.63.1.230-262.1999 PMC98963

[bib102] Wood JM . Bacterial osmoregulation: a paradigm for the study of cellular homeostasis. Annu Rev Microbiol. 2011;65:215–38. 21663439 10.1146/annurev-micro-090110-102815

[bib103] Yin W , Cai X, Ma H et al. A decade of research on the second messenger c-di-AMP. FEMS Microbiol Rev. 2020;44:701–24. 32472931 10.1093/femsre/fuaa019 PMC7850090

[bib104] Zhang P , Wu L, Zhu Y et al. Deletion of MtrA inhibits cellular development of *Streptomyces coelicolor* and alters expression of developmental regulatory genes. Front Microbiol. 2017;8:2013. 29085353 10.3389/fmicb.2017.02013 PMC5650626

